# Roles of constitutive and signal-dependent protein phosphatase 2A docking motifs in burst attenuation of the cyclic AMP response element-binding protein

**DOI:** 10.1016/j.jbc.2021.100908

**Published:** 2021-06-24

**Authors:** Sang Hwa Kim, Cheng-Guo Wu, Weiyan Jia, Yongna Xing, Randal S. Tibbetts

**Affiliations:** 1 Department of Human Oncology, University of Wisconsin-Madison School of Medicine and Public Health, Madison, Wisconsin, USA; 2 Department of Oncology, University of Wisconsin-Madison School of Medicine and Public Health, Madison, Wisconsin, USA

**Keywords:** CREB, PP2A, phosphorylation, Serine 133, cAMP, B56γ, PKA, SLiM, casein kinase, BS1/2, B56 binding site 1/2, CBP, CREB-binding protein, CK1/2, casein kinase 1/2, CLM, calicheamicin γ1, CREB, cAMP response element-binding protein, CRTCs, cAMP-regulated transcriptional coactivators, Fsk, Forskolin, KID, kinase-inducible domain, KIX domain, KID-interacting domain, PMA, Phorbol 12-myristate 13-acetate, PP2A, protein phosphatase 2A, SLiM, short linear motif

## Abstract

The cAMP response element-binding protein (CREB) is an important regulator of cell growth, metabolism, and synaptic plasticity. CREB is activated through phosphorylation of an evolutionarily conserved Ser residue (S133) within its intrinsically disordered kinase-inducible domain (KID). Phosphorylation of S133 in response to cAMP, Ca^2+^, and other stimuli triggers an association of the KID with the KID-interacting (KIX) domain of the CREB-binding protein (CBP), a histone acetyl transferase (HAT) that promotes transcriptional activation. Here we addressed the mechanisms of CREB attenuation following bursts in CREB phosphorylation. We show that phosphorylation of S133 is reversed by protein phosphatase 2A (PP2A), which is recruited to CREB through its B56 regulatory subunits. We found that a B56-binding site located at the carboxyl-terminal boundary of the KID (BS2) mediates high-affinity B56 binding, while a second binding site (BS1) located near the amino terminus of the KID mediates low affinity binding enhanced by phosphorylation of adjacent casein kinase (CK) phosphosites. Mutations that diminished B56 binding to BS2 elevated both basal and stimulus-induced phosphorylation of S133, increased CBP interaction with CREB, and potentiated the expression of CREB-dependent reporter genes. Cells from mice harboring a homozygous *Creb*^*E153D*^ mutation that disrupts BS2 exhibited increased S133 phosphorylation stoichiometry and elevated transcriptional bursts to cAMP. These findings provide insights into substrate targeting by PP2A holoenzymes and establish a new mechanism of CREB attenuation that has implications for understanding CREB signaling in cell growth, metabolism, synaptic plasticity, and other physiologic contexts.

The cyclic AMP response element (CRE)-binding protein (CREB) is a signal-regulated transcription factor that has served as a model for understanding phosphorylation-coupled folding, coactivator binding, and gene regulation (1). Conserved throughout metazoan evolution, CREB regulates cell growth, survival, and differentiation and plays instrumental roles in neuronal long-term potentiation, hepatic glucose metabolism, and other physiologic processes (2, 3).

The canonical CREB activation pathway involves its cAMP-inducible phosphorylation on S133 by protein kinase A (PKA) (4, 5). S133 is located within an ∼60 aa intrinsically disordered region (IDR) termed the kinase-inducible domain (KID) (6, 7). Phosphorylation of S133 promotes interaction of the KID with the KIX domain of the transcriptional coactivator, CBP, and its closely related paralog, p300 (CBP/p300) (8). Binding is initiated by transient weak interactions between the unstructured phospho-KID (pKID) and KIX that promote folding of pKID into orthogonal α-helices that mediate specific association (9, 10).

A second class of CREB coactivators—the cAMP-regulated transcriptional coactivators (CRTCs)—activates CREB independent of S133 phosphorylation (11, 12, 13, 14). CRTCs are retained in the cytoplasm as a consequence of phosphorylation by salt-inducible kinases (SIKs) and binding to 14-3-3 proteins (2). Cytoplasmic sequestration of CRTC2 is relieved by cAMP, which triggers PKA-dependent phosphorylation and inactivation of SIKs, or Ca^2+^, which stimulates the Ca^2+^-dependent phosphatase calcineurin to dephosphorylate CRTC2 (14, 15). Upon entering the nucleus, CRTCs bind the CREB bZIP domain and enhance both its DNA-binding activity and interaction with CBP/p300 (15, 16, 17, 18). The cooperative assembly of CREB-CBP/p300-CRTC ternary complexes is thought to underly potent induction of CREB target genes by cAMP and Ca^2+^ (17, 18).

CREB is also phosphorylated on S133 in response Ca^2+^ by calmodulin kinases II and IV (19, 20, 21), and by mitogen and stress-activated kinases 1 and 2 (MSK1/MSK2) in response to mitogens such as phorbol 12-myristate 13-acetate (PMA) and stress insults (22, 23). While these stimuli trigger S133 phosphorylation stoichiometries similar to those induced by cAMP and Ca^2+^, they are relatively weak inducers of CRTC nuclear accumulation and fail to robustly induce CREB-CBP ternary complexes over CRE-containing promoters (17, 18, 24). Nevertheless, an S133A mutation compromised PMA-inducible CREB target gene expression in mice, suggesting CBP/p300-independent roles for S133 in transcriptional activation (24). Other phosphorylation sites within the CREB KID are thought to function in a modulatory capacity. Phosphorylation of S142 is induced by circadian entrainment and many of the same stimuli that induce S133, yet this site antagonizes CREB-CBP interaction (25, 26, 27). Processive phosphorylation of S129 by GSK3 following PKA-dependent priming phosphorylation of S133 has been implicated in the inhibition CREB DNA-binding activity (28), though its stoichiometry and functional importance are uncertain.

Our laboratory has investigated a conserved cluster of Ser residues within the KID dubbed the CK cassette that undergoes processive phosphorylation by ataxia-telangiectasia mutated (ATM) and casein kinases 1 and 2 (CK1, CK2) in response to genotoxic stress (29, 30). Within this motif, ATM-dependent phosphorylation of S111 primes processive phosphorylation of S108 by CK2 and S114 and S117 by CK1. Phosphorylation of all four sites enables a fifth, ATM-dependent phosphorylation event on S121 (29). Although the biochemical implications of the CK cassette have not been fully elucidated, its phosphorylation in response to DNA damage modestly reduced interaction with the KIX domain of CBP (30). More recently, it was shown that ATM- and CK1/2-dependent phosphorylation of CK cassette inhibited CREB DNA-binding activity in a graded manner by promoting an electrostatic, autoinhibitory interaction with the bZip domain (31, 32). An inhibitory role for the CK cassette is consistent with the modest suppression of CREB transcriptional activity following cellular exposure to DNA damage (30, 31). Although the cellular implications are uncertain, ATM and CK-dependent inhibition of CREB likely contributes to adaptive responses to DNA damage. It is also conceivable that the CK cassette influences CREB activity in response to non-DNA-damaging stimuli.

Finally, while the kinases controlling CREB phosphorylation have garnered much attention, comparatively little is known regarding the mechanisms of CREB dephosphorylation. Both PP1 and PP2A have been implicated as S133 phosphatases (33, 34) while the CK cassette was reported to be dephosphorylated by PP2A through B56-type regulatory subunits (35).

In this study we show that the CK cassette and S133 are coregulated during canonical CREB activation by cAMP and that recruitment of PP2A-B56 holoenzymes to a constitutive B56-docking motif enhanced by an acidic patch triggers the dephosphorylation of S133 and the CK cassette. Cells expressing CREB with disruptive mutations in this motif exhibits increased S133 phosphorylation and transcription potential. These results highlight the interdependence of CREB phosphorylation mechanisms and establish new models for probing functional impacts of CREB signaling *in vivo*.

## Results

### The CREB CK cassette and S133 are coregulated by cAMP

**Figure 1:**
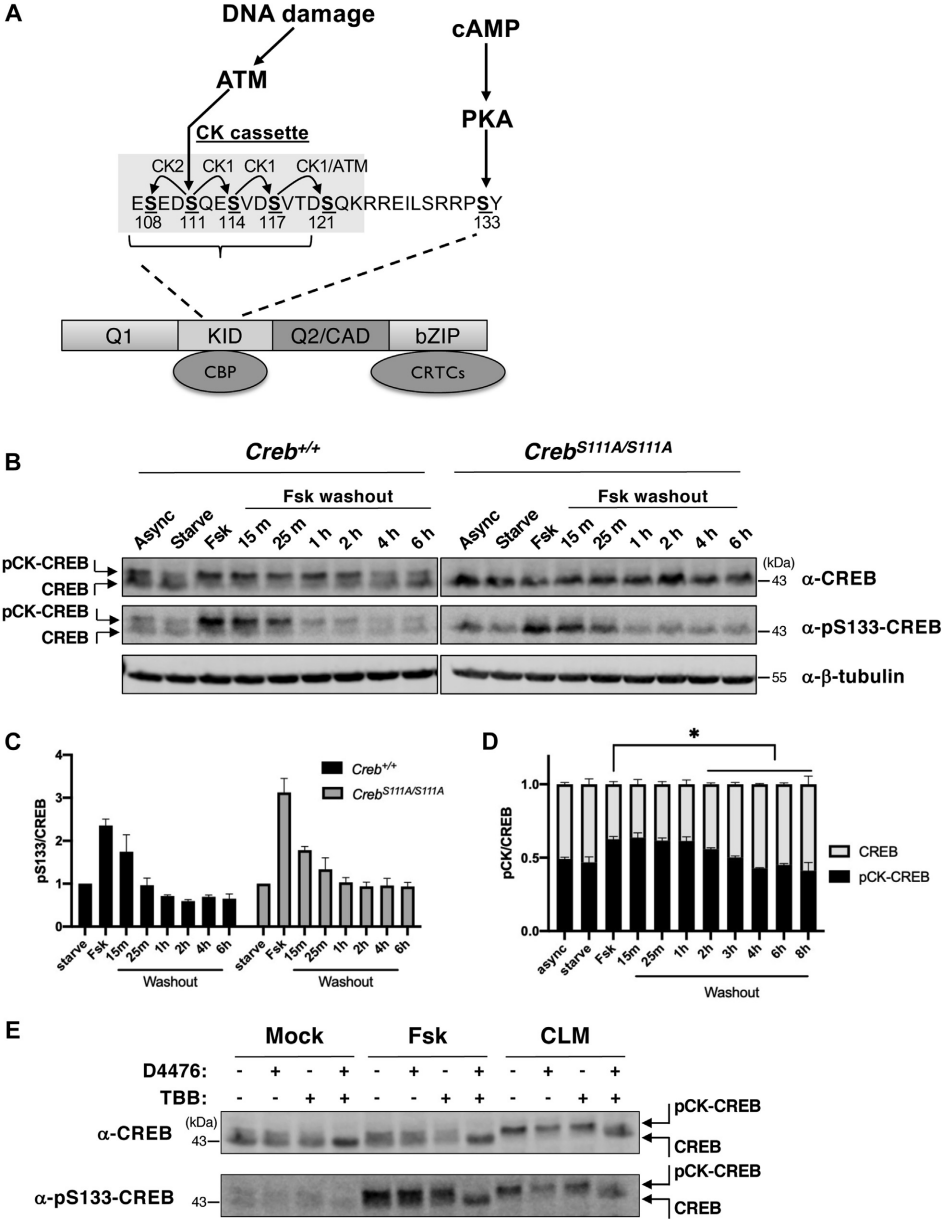
**The CREB CK cassette and S133 are coregulated by cAMP.***A*, CREB phosphoregulation by DNA damage and cAMP. CREB stick diagram and expanded view of the casein kinase (CK) cassette. ATM-dependent phosphorylation of S111 triggers processive phosphorylation of flanking Ser residues by CK1 and CK2. Approximate binding regions for CBP (CREB-binding protein) and CRTCs (CREB-regulated transcriptional coactivators) are shown, as are Q1 and Q2/CAD (constitutive activation domain) Gln-rich regions and the bZip DNA-binding domain. *B*, time course phosphorylation on CK cassette and S133 phosphorylation in response to Fsk. *Creb*^*+/+*^ and *Creb*^*S111A/S111A*^ MEFs were treated with Fsk for 30 min followed by Fsk washout for the indicated lengths of time. CK cassette phosphorylation manifested as an electrophoretic mobility shift that was absent in *Creb*^*S111A/S111A*^ MEFs while S133 phosphorylation was monitored with a pS133-specific antibody. Note coincident phosphorylation of the CK cassette and S133. *C*, quantification of pS133/CREB levels in *Creb*^*+/+*^ and *Creb*^*S111A/S111A*^ MEFs (n = 4). *D*, quantification of pCK/CREB levels in *Creb*^*+/+*^ MEFs (n = 4, ∗*p* < 0.05). *E*, Fsk-induced CK cassette phosphorylation requires CK1 and CK2. MEFs were treated with CK1 (D4476) and/or CK2 (TBB) inhibitors 30 min prior to treatment with Fsk or the radiomimetic agent CLM for 30 min. CK cassette and S133 phosphorylation monitored with the indicated antibodies.

Previous work established that, in addition to undergoing stoichiometric phosphorylation following DNA damage, the CK cassette is also phosphorylated independently of DNA damage (31) (Fig. 1*A*). To investigate the relationship between the CK cassette and canonical regulation of CREB by cAMP, we measured CREB phosphorylation in mouse embryo fibroblasts (MEFs) following a pulsatile exposure to forskolin (Fsk), which stimulates cAMP production. Phosphorylation of the CK cassette (pCK-CREB) was induced within 30 min of Fsk exposure as evidenced by a characteristic electrophoretic mobility shift that reflects CK1/2-dependent phosphorylation of S108, S111, S114, and S117 (Fig. 1*B*) (29). Washout experiments revealed that maximal CK cassette phosphorylation persisted for 60 min after Fsk removal, returning to baseline levels after an additional 4 to 6 h while S133 phosphorylation was relatively more labile, returning to baseline levels within 1 h of Fsk washout (Fig. 1, *B*–*D*). The phosphorylation and dephosphorylation profiles of S133 were comparable between *Creb*^*+/+*^ *and Creb*^*S111A/S111A*^ MEFs (Fig. 1, *B* and *C*) suggesting that phosphorylation of the CK cassette does not have a major influence on S133 kinases and phosphatases. Fsk-induced phosphorylation of the CK cassette was blocked by a combination of CK1 (D4476) and CK2 (TBB) inhibitors but not by either inhibitor alone (Fig. 1*E*). Consistent with previous findings, CK1/CK2 inhibition also suppressed phosphorylation of CREB in response to calicheamicin γ1 (CLM)-induced DNA damage (Fig. 1*E*). Finally, other agents that induce S133 phosphorylation, including PMA and serum, also increased the pCK-CREB/CREB ratio; however, CK cluster phosphorylation stoichiometry was lower than that seen in Fsk-treated cells (Fig. S1, *A* and *B*). From the combined findings we conclude that Fsk triggers coincident phosphorylation of S133 and the CK cassette while the dephosphorylation profiles of the two motifs differ.

### PP2A-B56 holoenzymes mediate CREB dephosphorylation

**Figure 2:**
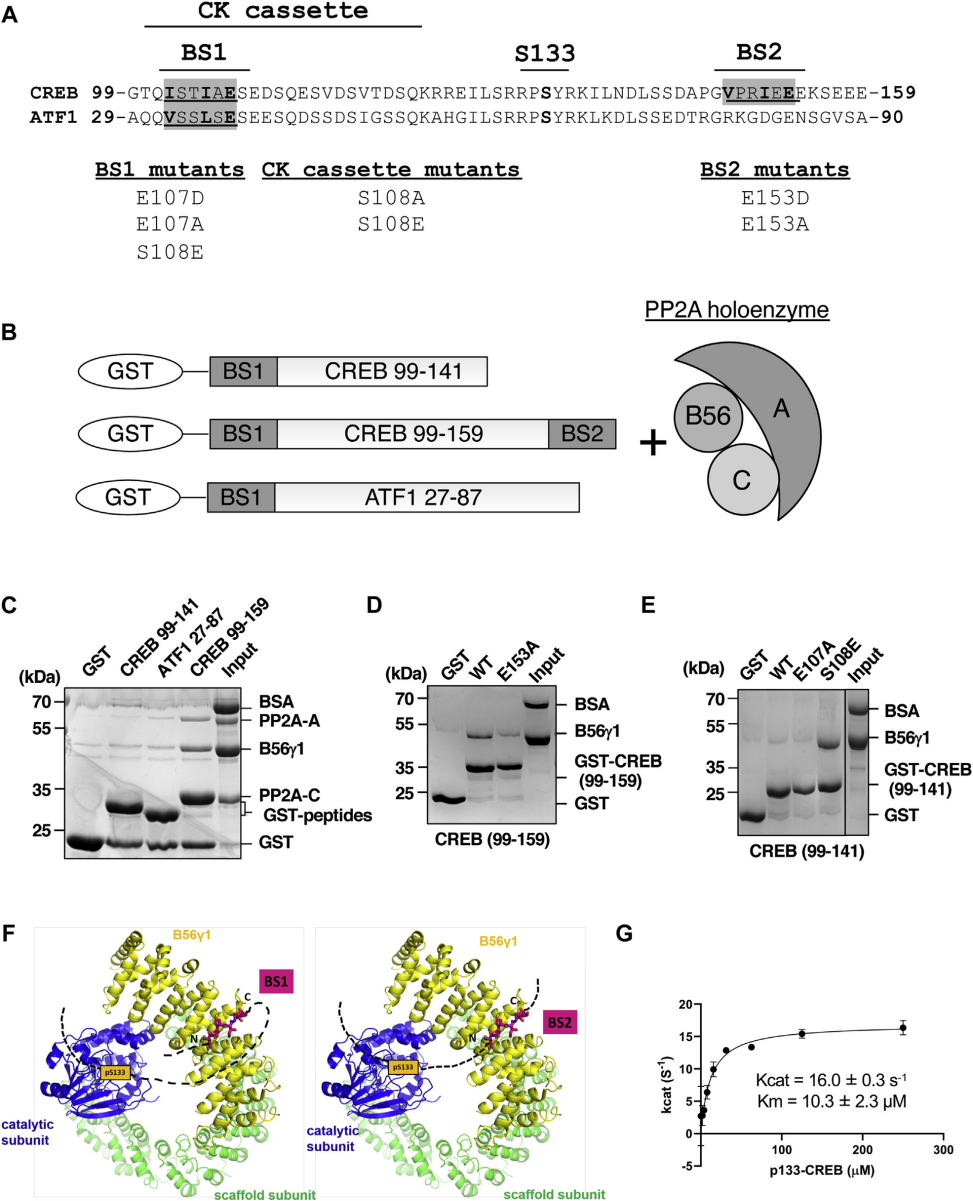
**Specific interactions and activities of PP2A-B56 subunits and holoenzyme toward CREB.***A*, consensus B56-binding sites in CREB and ATF1 in relation to the CK cassette and S133. Consensus B56 bindings sites (BS1 and BS2) are overlined and binding site and phosphosite mutants are indicated. *B*, schematic of GST-CREB and GST-ATF1 fusion proteins and PP2A holoenzyme used for pull-down assays. *C*, pull-down of PP2A-B56γ1 holoenzyme *via* GST-CREB (99–141), GST-CREB (99–159), or ATF1 (27–87) immobilized on GS4B resin. Bound proteins and input for pull-down were examined on SDS-PAGE and visualized by Coomassie blue staining. *D*, GST-CREB (99–159) bearing BS2 mutations. The bound proteins were examined as in *C*. *E*, assess the effects of BS1 mutations by pull-down of B56γ1 *via* GST-CREB (99–141) bearing BS1 mutations. *F*, structural models for the PP2A-B56γ1 holoenzyme bound to BS1 (*left*; *magenta sticks*) and BS2 (*right*; *magenta sticks*) and placement of pS133 (*orange box*) to the phosphatase active site. The *dashed lines* stand for peptide fragments. N and C stand for N-terminal and C- terminal sides of the bound motifs. *G*, steady-state enzyme kinetics of PP2A-B56γ1 holoenzyme phosphatase activity toward titrated concentrations of pS133-CREB (129–154). Kcat and Km were calculated.

Recognition of distinct short linear motifs (SLiMs) in substrates is an emerging theme for PP2A regulatory subunits (from four families, B/B55, B’/B56, B’’/PR72, and B’’’/PR93) to control substrate specificities of diverse PP2A holoenzymes. B55 and B56 subunits of PP2A were recently suggested to recognize a bipartite polybasic motif (36) and an LxxIxE motif, respectively (37, 38, 39, 40, 41, 42). Analysis of CREB amino acid sequence identified two potential B56-docking motifs: a site near the amino-terminus of the KID spanning residues 102 to 107 (binding site 1 (BS1)) and a site near the carboxyl-terminus of the KID spanning residues 148 to 153 (BS2) (Fig. 2*A*). Of these, only BS1 is positionally conserved in the CREB paralog ATF1 (Fig. 2*A*). Pull-down assays revealed that a GST-CREB (99–159) fusion protein containing both BS1 and BS2 interacted with B56γ1, a member from the B56 family, but not B55α, a member from B55 family (Fig. S2*A*). In contrast to CREB (99–159), CREB (99–141) containing only BS1 exhibited weak binding to B56γ1 in GST pull-down assays (Fig. 2, *B* and *C*, Fig. S2, *B* and *C*). CREB (126–159) containing only BS2 bound to B56γ1 as avidly as CREB (99–159) containing both BS1 and BS2, suggesting that BS2 is the dominant B56-binding motif (Fig. S2, *B* and *C*). Consistent with this notion, B56γ1 only weakly associated with the GST-ATF1 (Fig. 2*C*), and E153A, a mutation to the required residue for B56 binding within BS2, abolished CREB (99–159) binding to B56γ1 (Fig. 2*D*). Finally, the weak binding of B56γ1 to CREB (99–141) was potentiated by a phosphomimetic S108E mutation (Fig. 2*E*). This finding is consistent with studies showing that phosphorylation on second or seventh to ninth positions of B56-motifs enhanced the interactions between B56 subunits and their substrates (37, 38). Because S108 (corresponding to the +7 position of the B56-binding consensus) undergoes phosphorylation following DNA damage (29) and cAMP (Fig. 1*B*), these results suggest that BS1 might be a stimulus-dependent B56 recruitment motif.

Using crystal structures of the PP2A-B56γ1 holoenzyme (PDB code:2NPP) and B56γ1-BubR1 substrate peptide complex (PDB code:5JJA), we built structural models for the PP2A-B56γ1 holoenzyme bound to the upstream and downstream CREB motifs (Fig. 2*F*). These models reveal that BS2 is structurally favorable for placing pS133 to the phosphatase active site (Fig. 2*F*, right panel). In contrast, the binding of BS1 requires a sharp turn in CREB for pS133 to access the phosphatase active site (Fig. 2*F*, left panel). The lack of residues to make sharp turns in the spacer between BS1 and pS133 (Fig. 2*A*) makes this motif energetically ineffective to target the holoenzyme to pS133. Finally, we calculated Kcat (16.0/s) and Km (10.3 μM) of the PP2A-B56γ1 holoenzyme toward a phosphorylated pS133-CREB (129–154) peptide (Fig. 2*G*). Consistently, the affinity between CREB and B56γ1 was estimated to be between 7.5 and 15 μM by titration pull-down assay (Fig. S2, *D* and *E*). Taken together, the *in vitro* studies implicate BS2 as functional B56-binding motif while BS1 may function as a phosphorylation-dependent B56 recruitment domain.

### BS2 mediates CREB dephosphorylation in intact cells

To investigate functional implications of B56-PP2A binding to CREB, we generated a panel of FLAG-tagged CREB mutants in which BS1, BS2, or both motifs were disrupted through substitution of the critical +5 Glu residue with either Ala or Asp. We also introduced eight Ser/Thr to Ala substitutions into the CK cassette (see Experimental procedures) to examine potential impacts on S133 dephosphorylation kinetics.

**Figure 3:**
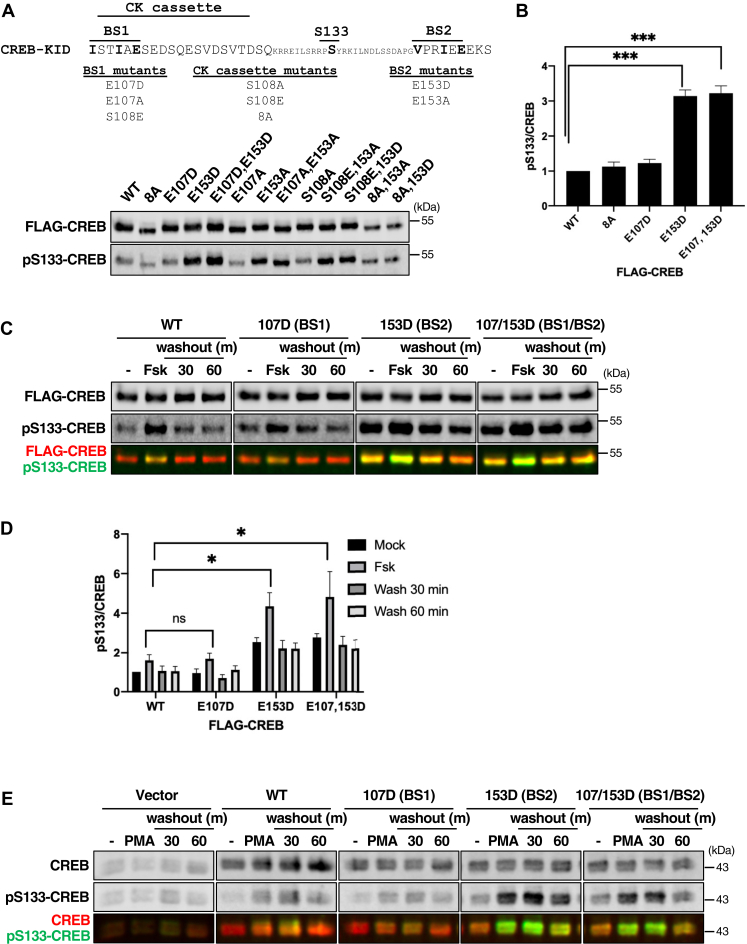
**Effect of B56-binding site mutations on CREB S133 dephosphorylation.***A*, indicated FLAG-tagged CREB proteins were expressed in HeLa cells for 72 h. Cell extracts were resolved by SDS-PAGE and coimmunoblotted for FLAG and pS133-CREB. *B*, quantification of pS133/FLAG-CREB levels in HeLa cells (n = 7, ∗∗∗*p* < 0.005). Panels *C* and *D* indicate FLAG-CREB proteins were expressed in HeLa cells, which were then treated with Fsk for 30 min before drug washout for the indicated lengths of time. Cell extracts were coimmunoblotted with α-FLAG and α-pS133-CREB antibodies. *D*, quantification of immunoblotting data from panel *C* (n = 4, ∗*p* < 0.05). *E*, untagged CREB proteins were expressed in HeLa cells, which were then treated with PMA for 30 min before drug washout for the indicated lengths of time. Cell extracts were coimmunoblotted with α-CREB and α-pS133-CREB antibodies.

Whereas BS1 mutations (E107D/A) did not appreciably affect pS133 levels, BS2 (E153D/A) mutations caused a 3- to 4-fold increase in baseline S133 phosphorylation (Fig. 3, *A* and *B*). pS133 levels were comparable between CREB^E153D^ and CREB^E107D/E153D^ double mutants suggesting that BS2 is the major regulator of S133 dephosphorylation in the absence of stimulation.

We also compared S133 phosphorylation levels between FLAG-tagged CREB^WT^, CREB^E107D^, and CREB^E153D^ following pulsatile exposure of HeLa cells to Fsk. CREB^E153D^ exhibited much higher level of both basal and induced phosphorylation relative to CREB^WT^ or CREB^E107D^ (Fig. 3, *C* and *D*). All three CREB proteins were dephosphorylated within 30 min after Fsk removal; however, residual levels of pS133 were higher for CREB^E153D^ than CREB^WT^ or CREB^E107D^ (Fig. 3, *C* and *D*). Similar findings were made using PMA-treated HeLa cells (Fig. 3*E*). These findings indicate that BS2 mutations increase basal and stimulus-induced CREB S133 phosphorylation; however, BS2 is not absolutely required for CREB dephosphorylation following stimulus removal.

Finally, because a phosphomimetic S108E mutation enhanced B56 binding to GST-CREB *in vitro* (Fig. 2*E*), we considered the possibility that an S108E mutation would accelerate S133 dephosphorylation following Fsk washout. However, the S133 dephosphorylation rate was comparable between CREB^WT^, CREB^S108A^, and CREB^S108E^ following Fsk removal (Fig. S3, *A* and *B*). These findings suggest that BS1 does not play a major role in the dephosphorylation of overexpressed CREB.

### Dephosphorylation of the CK cassette is partially dependent on BS2

**Figure 4:**
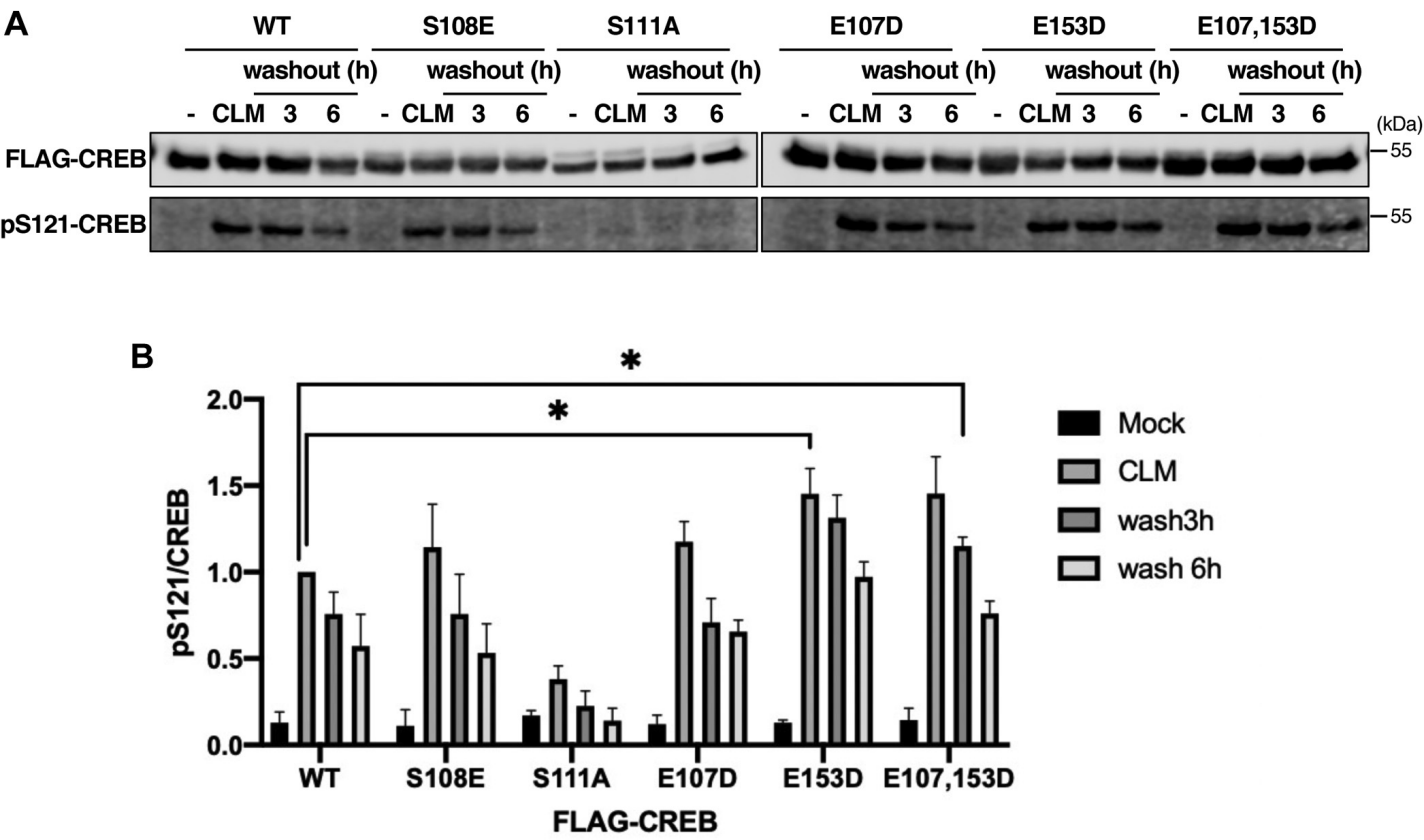
**Dephosphorylation of the CK cassette is partially dependent on BS2.***A*, the indicated FLAG-CREB proteins were expressed in HeLa cells for 48 h prior to treatment with 2 nM CLM for 30 min. Cells were harvested at the indicated times following CLM washout and extracts immunoblotted with the indicated antibodies. *B*, quantification of CREB dephosphorylation data (n = 3, ∗*p* < 0.05).

We also measured impacts of BS1 and BS2 mutations on CK cassette dephosphorylation kinetics. To this end, we measured S121 phosphorylation/dephosphorylation in HeLa cells expressing FLAG-tagged wild-type and BS1/BS2-mutant CREB proteins following pulsatile exposure to the radiomimetic agent CLM. Consistent with previous work, CLM strongly induced S121 phosphorylation, and this was virtually abolished by mutation of the critical CK1/2 site at S111 (29) (Fig. 4*A*). While the E107D (BS1) mutation had no discernible effect, E153D (BS2) and E107D/E153D (BS1/BS2) mutations modestly increased the proportion of CREB phosphorylated after CLM treatment (Fig. 4*B*). Meanwhile, the phosphomimetic S108E mutation did not significantly alter the magnitude or duration of S121 phosphorylation in CLM-treated HeLa cells. The combined findings suggest that, in addition to regulating S133 dephosphorylation, BS2 contributes to PP2A-dependent dephosphorylation of the CK cassette.

### BS2 mutations enhance CREB transcriptional potential

**Figure 5:**
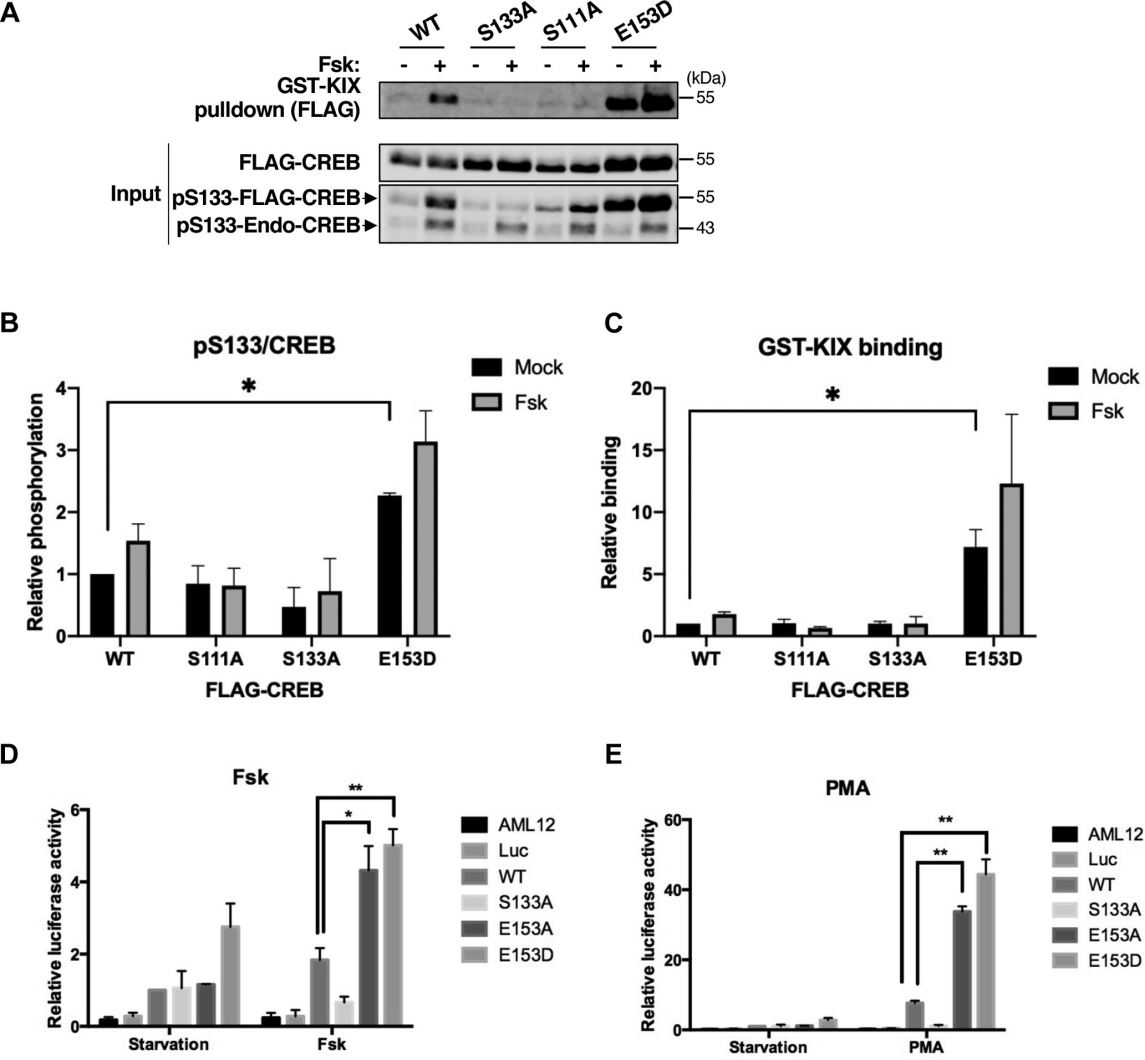
**Enhanced CBP binding and transactivation potential of CREB BS2 mutants.***A*, indicated FLAG-CREB proteins were expressed in HeLa cells and affinity purified *via* GST pull-down assays using the KIX domain of CBP. *B* and *C*, quantification of pS133/CREB levels (*B*) and GST-KIX pull-down efficiency (*C*) for the indicated CREB proteins (n = 3, ∗*p* < 0.05). *D* and *E*, AML-12 hepatocytes were cotransfected with indicated UAS-Luciferase (Luc) and Gal4-CREB plasmids. Cells were serum starved for 16 h and then treated for 2 h with Fsk (*D*) or PMA (*E*) prior to luciferase activity measurements (n = 3, ∗*p* < 0.05; ∗∗*p* < 0.01).

To determine whether BS2 mutations potentiate CREB-dependent transcription, we first measured binding of CREB^WT^, CREB^E107D^ (BS1), and CREB^E153D^ (BS2) to a GST fusion with the KID-interacting (KIX) region of CBP. Consistent with its increased S133 phosphorylation (Fig. 5, *A* and *B*), CREB^E153D^ showed enhanced Fsk-inducible binding to the GST-KIX to relative to CREB^WT^ while an S133A mutation abolished binding (Fig. 5, *A* and *C*). Interestingly, disruption of CK cassette phosphorylation *via* the S111A mutation also significantly reduced Fsk-inducible binding of CREB to GST-KIX. This finding is consistent with the modest CREB activation defect previously seen in CREB^S111A^ cells (31).

We next measured transactivation potential of wild-type and mutant Gal4-CREB fusions toward a UAS-luciferase reporter gene in AML-12 hepatocytes. While CREB^S133A^ was transcriptionally inert, CREB^E153A^ and CREB^E153D^ showed enhanced activity in response to Fsk (Fig. 5*D*) or PMA (Fig. 5*E*). We conclude that inhibition of B56-PP2A binding enhances CBP binding and CREB transactivation potential.

A previous study identified a CREB^Y134F^ mutant that exhibited more efficient phosphorylation by S133 kinases, including PKA (43). Reasoning that Y134F and E153D mutations would lead to synergistic impacts on S133 phosphorylation, we measured pS133 levels of FLAG-tagged CREB^WT^, CREB^E153D^, and CREB^Y134F/E153D^ proteins expressed in HeLa cells. Somewhat surprisingly, baseline and PMA-induced S133 phosphorylation were comparable between CREB^WT^ and CREB^Y134F^, while phosphorylation of CREB^E153D^ was elevated 3 to 4 fold over both CREB^WT^ and CREB^Y134F^ (Fig. S4, *A* and *B*). Although the Y134F mutation could plausibly diminish CREB recognition by pS133 antibodies, this is unlikely since the CREB^Y134F/E153D^ double mutant showed ∼3-fold upregulation of pS133 phosphorylation relative to CREB^Y134F^ (Fig. S4, *A* and *B*). Similar findings were made using transfected HeLa cells stimulated with Fsk (Fig. S4, *C* and *D*). On the other hand, the Y134F mutation increased Gal4-CREB transactivation potential to a greater extent than the E153D mutation and the activity of a combined Gal4-CREB^Y134F/E153D^ mutant was comparable to the Gal4-CREB^Y134F^ mutant (Fig. S4*E*). These findings suggest that the Y134F mutation activates CREB independent of increases in S133 phosphorylation stoichiometry and is dominant to the E153D mutation in transcriptional reporter assays.

To test whether the transcriptional enhancement by BS2 mutations was due to increased S133 phosphorylation stoichiometry, we measured transactivation potential of Gal4-CREB fusions harboring S133A and E153D mutations. Baseline transcriptional activities of Gal4-CREB^E153D^ and Gal4-CREB^S133A/E153D^ were comparably elevated over the activity of Gal4-CREB^WT^ (Fig. S5*A*), suggesting an S133-independent effect of BS2 on CREB transactivation potential. By contrast the S133A mutation abolished the inducible component of Gal4-CREB^E153D^ activity in HeLa cells treated with Fsk (Fig. S5*A*) or PMA (Fig. S5*B*). As expected, the reduced transactivation potential of CREB^S133A/E153D^ relative to CREB^E153D^ correlated with reduced inducible binding to the CBP KIX domain (Fig. S5, *C* and *D*). We conclude that BS2 mutations potentiate inducible CREB transactivation and CBP binding through a pS133-dependent mechanism.

### BS2 attenuates S133 phosphorylation and CREB transcriptional activation *in vivo*

To ascertain physiologic implications of CREB dephosphorylation by B56-PP2A, we engineered an E153D mutation into the mouse *Creb* locus using CRISPR/CAS9 (see Experimental procedures). Two *Creb*^*E153D*^ founder lines were used to generate homozygous *Creb*^*E153D/E153D*^ mutant mice that were viable, fertile, and born at the predicted Mendelian frequency (not shown). A full phenotypic description of *Creb*^*E153D*^ mice will be presented elsewhere.

**Figure 6:**
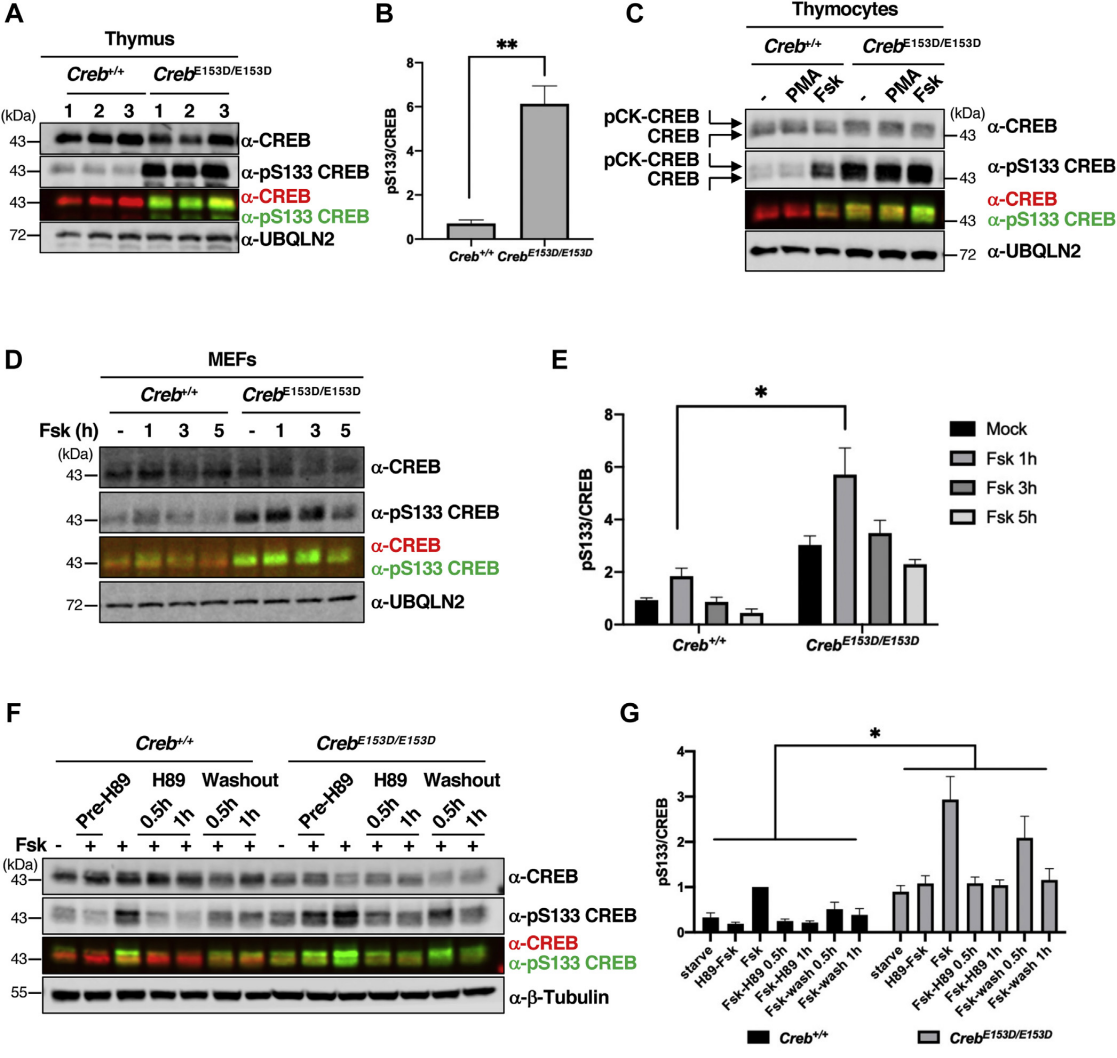
**An endogenous E153D mutation potentiates CREB phosphorylation.***A*, thymus extracts were prepared from 8-week-old *Creb*^*+/+*^ or *Creb*^*E153D/E153D*^ mice. Three different thymi from each genotype were coimmunoblotted for total CREB and pS133-CREB. *B*, quantification of immunoblotting data in panel *A* (n = 3, ∗∗*p* < 0.01). *C*, *ex vivo* cultured *Creb*^*+/+*^ or *Creb*^*E153D/E153D*^ thymocytes were treated with PMA or Fsk for 1 h and immunoblotted for total CREB and pS133-CREB. *D*, *Creb*^*+/+*^ or *Creb*^*E153D/E153D*^ MEFs were treated with Fsk for the indicated time and total CREB and pS133-CREB were analyzed be immunoblotting. *E*, quantification of pS133/CREB levels in *Creb*^*+/+*^ and *Creb*^*E153D/E153D*^ MEFs (n = 3, ∗*p* < 0.05). *F*, *Creb*^*+/+*^ or *Creb*^*E153D/E153D*^ MEFs were treated with Fsk for 30 min prior to H89 treatment (20 μM) or drug washout for the indicated lengths of time. Cell extracts were coimmunoblotted with α-CREB and α-pS133-CREB antibodies. *G*, quantification of pS133/CREB levels in *Creb*^*+/+*^ and *Creb*^*E153D/E153D*^ MEFs (n = 3, ∗*p* < 0.05).

Baseline S133 phosphorylation was elevated in the thymus, spleen, cerebrum, and cerebellum of *Creb*^*E153D/E153D*^ mice relative to *Creb*^*+/+*^ mice (Fig. 6, *A* and *B*, Fig. S6, *A*–*C*). The fraction of CK1/2-phosphorylated CREB was also elevated in *Creb*^*E153D/E153D*^ thymocytes relative to *Creb*^*+/+*^ thymocytes, supporting the idea that BS2 mediates dephosphorylation of the CK cassette (Fig. 6*C*). Basal and Fsk-induced phosphorylation of S133 were significantly upregulated in primary *Creb*^*E153D/E153D*^ MEFs relative to *Creb*^*+/+*^ MEFs (Fig. 6, *D* and *E*).

To evaluate how the endogenous E153D mutation influences the rate of CREB dephosphorylation, we compared pS133 levels between *Creb*^*+/+*^ and *Creb*^*E153D/E153D*^ MEFs that had been treated by Fsk for 30 min followed by washout or treatment with PKA inhibitor, H-89. As previously observed, the S133 phosphorylation maximum was elevated in *Creb*^*E153D/E153D*^ MEFs relative to *Creb*^*+/+*^ MEFs. H-89 treatment reduced pS133 levels in *Creb*^*+/+*^ and *Creb*^*E153D/E153D*^ MEFs with similar kinetics; however, residual pS133 levels were higher in *Creb*^*E153D/E153D*^ MEFs relative to *Creb*^*+/+*^ MEFs (Fig. 6, *F* and *G*). Qualitatively similar results were obtained following Fsk washout (Fig. 6, *F* and *G*). The combined findings support the role of BS2 in the phosphoregulation of endogenous CREB.

**Figure 7:**
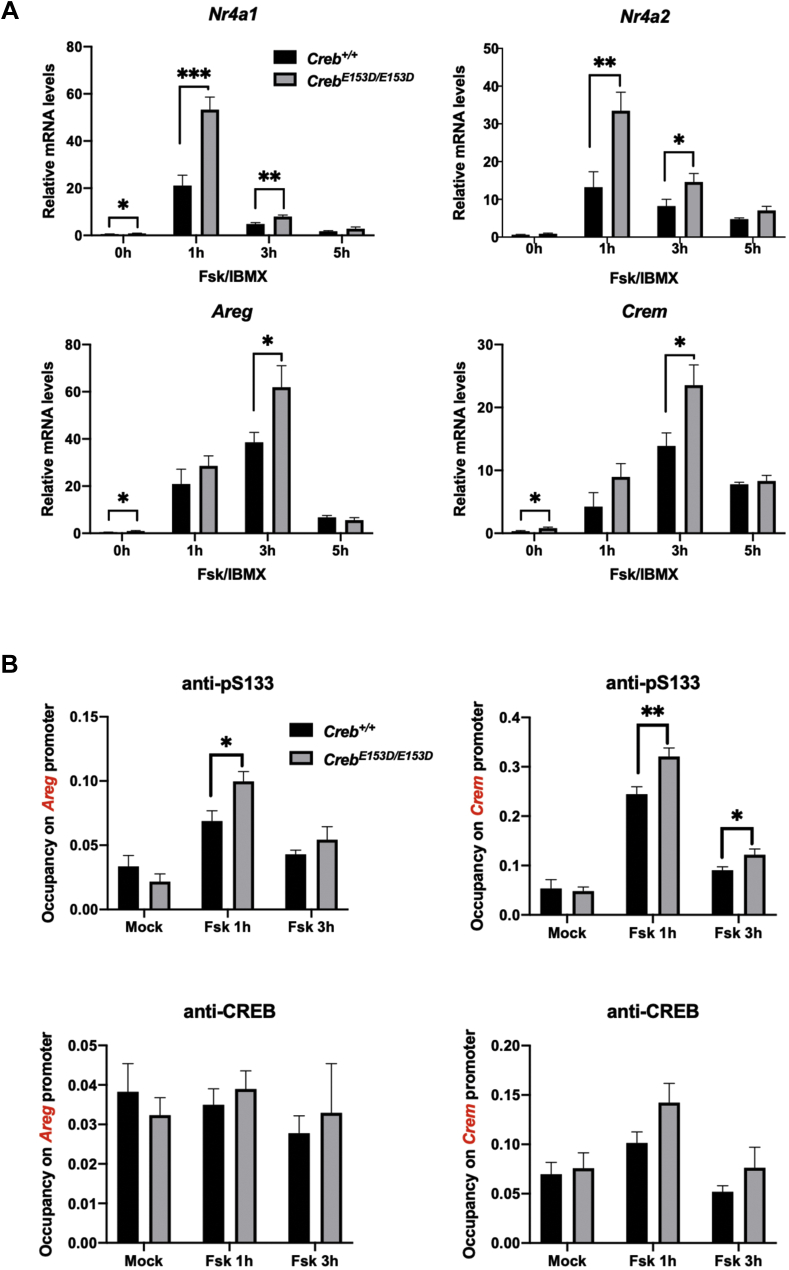
**BS2 regulates CREB transcriptional activity.***A*, *Creb*^*+/+*^ and *Creb*^*E153D/E153D*^ MEFs were treated with Fsk for the indicated time and levels of indicated genes determined by qPCR. Each bar represents the averaged results of seven (*Creb*^*E153D/E153D*^) or eight (*Creb*^*+/+*^) independently generated MEF cultures (n = 8, ∗*p* < 0.05; ∗∗*p* < 0.01; ∗∗∗*p* < 0.005). *B*, impact of E153D mutation on pS133-CREB occupancy over the *Areg* and *Crem/Icer* gene locus. MEFs of the indicated genotypes were treated with Fsk for the indicated time and processed for ChIP-qPCR using the indicated antibodies. Y-axis represents indicated gene promoter occupancy. Each bar represents averaged results (n = 7, Error bars indicate SEM. ∗*p* < 0.05).

We next measured the effects of the *Creb*^*E153D*^ mutation on the expression of a panel of CREB target genes. Fsk-inducible of the immediate-early genes *Nr4a1* and *Nr4a2* was significantly elevated in *Creb*^*E153D/E153D*^ MEFs relative to *Creb*^*+/+*^ MEFs, with the effect being most pronounced 1 h post stimulation, when *Nr4a1* and *Nr4a2* levels reached their maxima (Fig. 7*A*). Maximum induced levels of *Areg* and *Crem* were also significantly elevated in *Creb*^*E153D/E153D*^ MEFs relative to *Creb*^*+/+*^ MEFs following stimulation with Fsk for 3 h. By contrast, *Areg* and *Crem* expressions were not significantly different between *Creb*^*E153D/E153D*^ and *Creb*^*+/+*^ MEFs at a 1 h timepoint during the inductive phase of the Fsk response. Finally, as predicted, chromatin immunoprecipitation (ChIP) experiments revealed increased pS133-CREB occupancy over a CREB-binding site within the *Areg* and *Crem* gene promoter (Fig. 7*B*). These findings suggest that increased S133 phosphorylation stoichiometry leads to upregulation of CREB target gene expression in response to Fsk.

## Discussion

**Figure 8:**
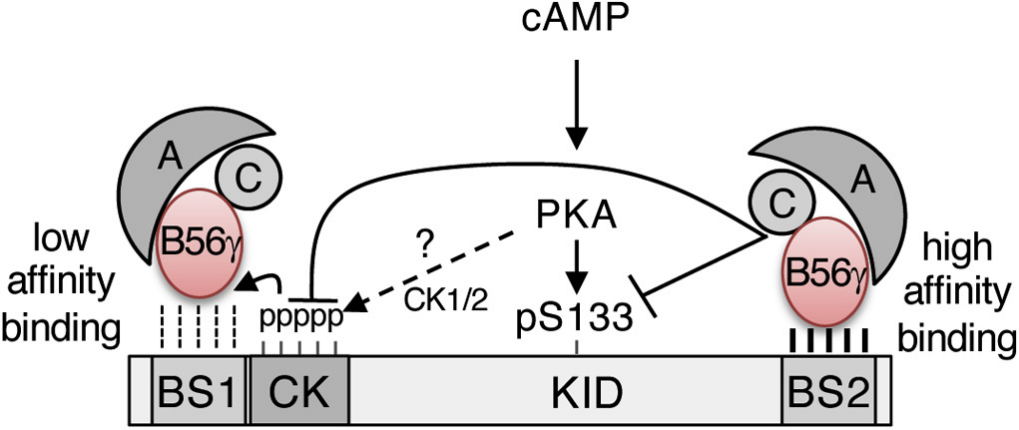
**Model for CREB dephosphorylation by PP2A.** cAMP induces PKA-dependent phosphorylation of S133 and stimulates CK1/2-dependent phosphorylation of the CK cassette through an undefined mechanism. High affinity, constitutive binding of B56γ1 to BS2 recruits PP2A catalytic (C) and regulatory (A) subunits to dephosphorylate S133 and the CK cassette (CK). Low-affinity binding of B56γ1 to BS1 may be enhanced by phosphorylation of the CK cassette; however, the Ser/Thr residues targeted for dephosphorylation by PP2A bound to this site are unclear.

In this study we have shown that CREB harbors functional B56-binding sites that facilitate PP2A-dependent dephosphorylation of S133, the CK cassette, and possibly other Ser/Thr residues within the CREB polypeptide. A schematic model summarizing our findings is presented in Figure 8. These findings support and extend previous work implicating PP2A as a CREB phosphatase (33, 35) and raise the possibility that regulated dephosphorylation is an important feature of the CREB activation paradigm.

*In vitro* binding studies and functional experiments using overexpressed or endogenous CREB^E153D^ proteins implicate BS2 as a major mediator of S133 and CK cassette dephosphorylation (Figs. 3, *A*–*D*, 4 and 5). BS2 mutations increased baseline and maximal phosphorylation levels of S133 without dramatically impacting dephosphorylation kinetics upon stimulus removal. The reason for this is unknown, but it is likely that PP2A, PP1 (34), and possibly other phosphatases redundantly dephosphorylate CREB independent of BS2 upon stimulus removal. Interestingly, BS2 is proximal to a Ser residue (S156) that is phosphorylated by CK1/2 *in vitro* (32), raising the possibility that S156 phosphorylation influences PP2A-B56 recruitment. Finally, although our studies focused on S133 and the CK cassette, it is also plausible that BS2 mediates the dephosphorylation of other Ser/Thr residues within KID, including functionally important S142 (25).

BS2 mutations enhanced CREB transcriptional potential ∼2 to 3 fold in reporter assays using Gal4-CREB fusions lacking C-terminal bZip and CRTC-binding domains (Fig. 5, *D* and *E*). While the effect was modest, Fsk-induced expression of a panel of CREB target genes was elevated in *Creb*^*E153D/E153D*^ MEFs relative to *Creb*^*+/+*^ MEFs (Fig. 7*A*). The more limited impact of the E153D mutation on endogenous gene expression likely reflects the contributions of CRTC-dependent CREB coactivation, the influence of CREB paralogs, and other transcription factors whose activity may be induced by cAMP. It is also possible that cellular CBP/p300 levels become limiting at levels of high S133 phosphorylation stoichiometry.

The functional implications of the low affinity B56-binding site, BS1, remain to be determined. Given that cAMP-induced phosphorylation of S108 within the CK cassette creates a favorable environment for B56 binding, we initially hypothesized that BS1 acts as a phosphorylation-dependent B56 recruitment domain. Consistent with this, a phosphomimetic S108E mutation stimulated the weak B56-binding activity of BS1 *in vitro* (Fig. 2*E*). However, CREB proteins harboring disruptive E107D/A mutations or the S108E phosphomimetic mutation exhibited S133 phosphorylation/dephosphorylation rates similar to those seen for wild-type CREB when expressed in HeLa cells (Fig. 3*C* and Fig. S3, *A* and *B*). This suggests that either BS1 is not functional or that B56-PP2A bound through BS1 mediates the dephosphorylation of different Ser/Thr residues on CREB or an associated factor.

Finally, it is interesting to consider the interplay between the CK cassette, S133, and BS2 in the context of tunable CREB-dependent gene regulation. While BS2 mutations potentiated S133 phosphorylation, they also increased phosphorylation of the CK cassette, which inhibits CREB DNA binding activity at high phosphorylation stoichiometries (31, 32). Although the biochemical mechanism is unclear, the coupling of S133 phosphorylation to inhibitory phosphorylation of the CK cassette may have evolved as a feedback mechanism to restrain CREB activity in the presence of prolonged stimulus. Future studies will define the interplay between activating and inhibitory inputs and assess whether CREB-B56-PP2A interactions are subjected to signal-dependent regulation.

## Experimental procedures

### Cell culture and treatments

MEFs were isolated from 14.5-day *Creb*^*+/+*^ and *Creb*^*E153D/E153D*^ embryos. Primary MEFs and HeLa cells were maintained in Dulbecco's modified Eagle's medium (DMEM) containing 10% fetal bovine serum (FBS) and 1% penicillin-streptomycin. Forskolin (Fsk, 10 μM, Sigma), 3-isobutyl-1-methylxanthine (IBMX, 100 μM, Sigma) or phorbol 12-myristate 13-acetate (PMA, 200 ng/ml, Sigma), H-89 (PKA inhibitor, 20 μM, Sigma) were added to cells for indicated times after overnight serum starvation. CLM (2 nM working concentration) was a kind gift of the Pfizer Compound Transfer Program and was maintained as a 4 μM stock solution in DMSO. CK1 (D4476) and CK2 (TBB) inhibitors were added 30 min prior to Fsk treatment and were used at concentrations of 20 μM and 50 μM, respectively. Gal4-CREB luciferase assays were performed using transiently transfected AML-12 hepatocytes (CRL-2254, ATCC) essentially as described (31).

### Molecular cloning and protein preparation

All constructs were generated using a standard PCR-based cloning strategy. Cloning, expression, and purification of full-length Aα, Cα, and B56γ1 subunits of PP2A and the preparation of PP2A-B56 holoenzymes followed the procedures described in previous studies (40, 44). Briefly, PP2A Aα and B56γ1 subunits were cloned into pGEX-2T and pGEX-6P vectors (GE Healthcare) and overexpressed in *Escherichia coli* strain BL21(DE3) for 16 h at 20 °C. The soluble fraction of the *E. coli* cell lysate was purified over GS4B resin (GE Healthcare) and further fractionated by ion exchange chromatography (Source 15Q, GE Healthcare). His-tagged PP2A Cα subunit (1–309) was cloned into a pFastBac vector (Invitrogen) and transformed into DH10Bac competent cells (Thermo Fisher Scientific) for bacmid preparation followed by baculovirus production. Hi-5 suspension cells (Thermo Fisher Scientific) grown to a density of 1.5 × 10^6^ cell/ml were infected with Cα baculovirus to express the protein. Cells were harvested and lysed after 48 h of infection. The soluble fraction of cell lysates was purified by Ni-NTA (QIAGEN) resin and further fractionated by Source 15Q. To assemble PP2A-B56γ1 holoenzyme, Cα subunit was first assembled with GST-Aα followed by the removal of GST- and His-tags using TEV protease. The protein mixture was further fractionated by Source 15Q to obtain pure AC dimer. Similar stochiometric amount of B56γ1 and AC dimer was then incubated in RT for 20 min and purified by gel filtration using a Superdex-200 column (GE Healthcare). WT and mutated GST-tagged CREB were cloned into pQLinkG vector (Addgene). The proteins were overexpressed 14 h at 23 °C in *E. coli* strain BL21 (DE3). The soluble fraction of the *E. coli* cell lysates was purified over GS4B resin and further fractionated by Source 15Q.

Gal4-CREB (pFA2-CREB; Agilent) encodes the 147 aa Gal4 DNA-binding domain fused to CREB amino acids 1 to 280. Expression of untagged and FLAG-tagged CREB from pFLAG-CMV and pcDNA3.1-based vectors was carried out as described (20, 24, 25). Protein was extracted from cultured cells in high-salt lysis buffer (25 mM HEPES (pH 7.4), 300 mM NaCl, 1.5 mM MgCl_2_, 1 mM EGTA, 0.1% Triton X-100) supplemented with protease inhibitors and phosphatase inhibitors for 10 min on ice, and proteins were separated on an SDS–polyacrylamide gel. Antibodies used in this study include: α-pS121-CREB (20), α-pS133-CREB (Cell Signaling, 87G3), α-CREB (Cell Signaling, 86B10), α-FLAG (Sigma, F7425), α-UBQLN2 (Cell Signaling, D7R2Z), and α-β-tubulin (Millipore, AA2). Site-specific mutagenesis was performed using the QuickChange PCR method. The CREB^8A^ construct was generated by replacing a *Bsp*EI-*Kpn*I fragment of the wild-type CREB coding sequence with a synthetic Gene Block DNA fragment (IDT) encoding Ala substitutions at Thr-100, Ser-103, Thr-104, Ser-108, Ser-111, Ser-114, Ser-117, and Ser-121.

### GST-mediated pull-down assay

Twelve micrograms of wild-type or mutated GST-tagged CREB (99–141, 99–159 or 126–159) was bound to 5 μl of glutathione resin *via* the GST tag. The resin was washed with 100 μl assay buffer containing 25 mM Tris (pH 8.0), 150 mM NaCl, and 2 mM DTT for three times to remove the unbound protein. To examine the interaction between CREB and B subunits of PP2A, 100 μg of Bα, or B56γ1 was added to the immobilized GST-CREB in a 50 μl of assay buffer supplemented with 1 mg/ml of BSA to avoid nonspecific binding. After 30 min of incubation, the unbound proteins were removed, and the resin was washed three times using 100 μl assay buffer supplemented with 0.1% Triton-X 100. The proteins remaining bound to resin were examined by SDS-PAGE and visualized by Coomassie blue staining. GST without CREB was used as control. A similar procedure was used to examine the binding affinity between B56γ1 and CREB except for that the immobilized WT GST-CREB (99–159) was incubated with titrated concentrations of B56γ1 for the pull-down assay. GST-KIX pull-down assays of transfected CREB proteins were performed as previously described (31).

### Phosphate sensor phosphatase assay

A phosphorylated CREB peptide containing CREB residues 129 to 154 with a phosphorylation on Ser133 was purchased from Genescript and used as the substrate for the enzyme kinetic assay. 10 nM of PP2A-B56γ1 holoenzyme mixed with 2× phosphate sensor (PV4406, Thermo Fisher Scientific) in 10 μl assay buffer containing 25 mM Tris HCl (pH 8.0), 150 mM NaCl, and 50 μM MnCl_2_ were dispensed in a 384-well, low-volume, round-bottom plates (Corning P/N 3677). Ten microliter of titrated concentrations (twofold dilutions from 250 μM to 3.9 μM) of the substrate prepared in assay buffer and a buffer control were then added to each well. Immediately, the microplate with samples was read by a plate reader at excitation 485 nm and emission 530 nm in the kinetic mode to collect data every 10 s for a total of 10 min. The steady-state kinetics of the PP2A-B56γ1 holoenzymes were determined by fitting the Michaelis–Menten equation (Equation 1) to the initial velocity of the dephosphorylation using various concentrations of the CREB peptide.

(1) $$V=\frac{K_{cat}\left[E\right]\left[S\right]}{K_{m}\;+\;\left[S\right]}$$

In Equation 1, kcat is the rate constant, [E] and [S] are enzyme and substrate concentrations, and Km is the Michaelis–Menten constant reflecting the binding affinity between the peptide substrate and the enzyme.

### Mouse strains

We employed CRISPR/CAS9 to introduce an E153D (BS2) mutation into the C57BL/6 mouse germline. A preferred gRNA target (GACTTTTCTTCTTCAATCCTTGG) was identified 9 bp upstream of codon 153 in the mouse *Creb1* gene using the MIT CRISPR Design Tool. The aggregate gRNA score of 58 was considered suitable for gene targeting. Four possible off-target cleavage sites with a cutting frequency determination (CFD) score of >0.5^43^ were identified using GUIDE-Seq (Gm26046-Trpd5213; Gm15297-Hmgn216; Klhl32 (intron); Gm13518-mmu-mir-195b). Three of these are intergenic and none are in linkage disequilibrium with the *Creb1* locus. Briefly, two independent *Creb*^*E153D*^ founder lines were obtained and yielded identical results. Tail samples were taken at weaning, and the targeted region was characterized using targeted ultradeep sequencing. The targeted region was PCR amplified (5;-GCTAGTTTGGTAAATGGGGGTTGGCACTGTTACAGTGGTGATGGCAGGGGCTGAAGTCTCCTCTTCTGACTTTTCgTCTTCgATCCTTGGCACCCCTGGTGCATCAGAAGATAAGTCATTCAAAATTTTCCTAGAAAAATAAAGAGCTATTTTAATTTT-3′) samples were indexed and pooled, and the pool was sequenced on a MiSeq 2 × 250 Nano. Resultant sequences were quality filtered, trimmed, and analyzed with CRISPResso (https://www.nature.com/articles/nbt.3583). Founders were backcrossed to B6J, unedited mice, and F1s were characterized similarly.

Heterozygous parents were used to give rise to experimental mice, allowing litters to contain a mix of genotypes. Thus, *Creb*^*+/+*^ and *Creb*^*E153D/E153D*^ mice from the same litters were used for all subsequent experiments.

The study was carried out in accordance with the recommendations in the Guide for the Care and Use of Laboratory Animals of the National Institutes of Health. The animal protocol was approved by the Campus Animal Care and Use Committee of the University of Wisconsin, Madison.

### Gene expression analysis and chromatin immunoprecipitation

RNA analysis was carried out as described (30). The gene-specific primers for qPCR were used: *Crem* (5′-TGATTCGCATAAACGTAGAGAAATTC-3′; 5′-CCATGGTAGCAATGTTAGGTGG-3′), *Areg* (5′- CACCATAAGCGAAATGCCTTC-3′; 5′-TCTTGGGCTTAATCACCTGTTC-3′), *Nr4a1* (5′-GCCTAGCACTGCCAACTTG-3′; 5′-TCTGCCCACTTTCGGATAAC-3′), *Nr4a2* (5′- GCGGAGACTTTAGGTGCATGT-3′; 5′- TTGTTTATGTGGCTTGCGC-3′), and *Gapdh* (5′-GCCTTCCGTGTTCCTACC-3′; 5′-CCTCAGTGTAGCCCAAGATG-3′).

Primary MEFs were cross-linked using 1% formaldehyde (Sigma) for 15 min, lysed and sonicated, and chromatin was immunoprecipitated with α-CREB (Cell Signaling, 86B10) and α-pS133 CREB (Cell Signaling, 87G3). After washing, the immunoprecipitated chromatin was reverse cross-linked and the liberated DNA fragments were purified using Qiagen spin columns. ChIP samples were measured by quantitative real-time PCR using the CFX96 Touch Real-Time PCR Detection System (Bio-Rad) and SYBR Green. Primer sequences for the promoters: ChIP-m*Crem* (5′-TGCTAGTTCTTTCCTCCTGCC-3′; 5′-CTCGGAGCTGACGTCAATGT-3′), ChIP-m*Areg* (5′-CGCGTAATCAGGGCTCGAT-3′; 5′-GTGGAGGCGGGAGGTTACTAC-3′). The results were expressed as the relative fold occupancy of the target precipitation as compared with the input.

### Statistical analysis

Statistical analysis was performed using the unpaired, two-tailed Student’s *t* test contained in Prism8 software. Data are presented as the mean ± standard error (SEM) from minimum three independent experiments. Differences were considered significant with ns: *p* > 0.05, ∗*p* < 0.05, ∗∗*p* < 0.01, ∗∗∗*p* < 0.001, and ∗∗∗∗*p* < 0.0001.

## Data availability

All data have been included within the manuscript.

## Supporting information

This article contains supporting information.

## Conflict of interest

The authors declare that they have no conflicts of interest with the contents of this article.
