# Supplementary material for: Roles of constitutive and signal-dependent protein phosphatase 2A docking motifs in burst attenuation of the cyclic AMP response element-binding protein
Source: J Biol Chem. 2021 Jun 24;297(1):100908. doi: 10.1016/j.jbc.2021.100908 (PMC8294589; doi:10.1016/j.jbc.2021.100908)
Supplement: Supplemental Figures S1–S6 [file mmc1.pdf]

Roles of constitutive and signal-dependent protein phosphatase 2A docking motifs in burst-attenuation of the cyclic AMP response element binding protein

Sang Hwa Kim[1], Cheng-Guo Wu[2], Weiyan Jia[1], Yongna Xing[2] and Randal S. Tibbetts[1]*

[1]Department of Human Oncology, [2]Department of Oncology, University of Wisconsin-Madison School of Medicine and Public Health, Madison, WI 53705

* To whom correspondence should be addressed.
Tel: 1-608-262-0027; Fax: 1-608-262-3913; Email: rstibbetts@wisc.edu

Running title: SLiMs mediate CREB dephosphorylation by PP2A

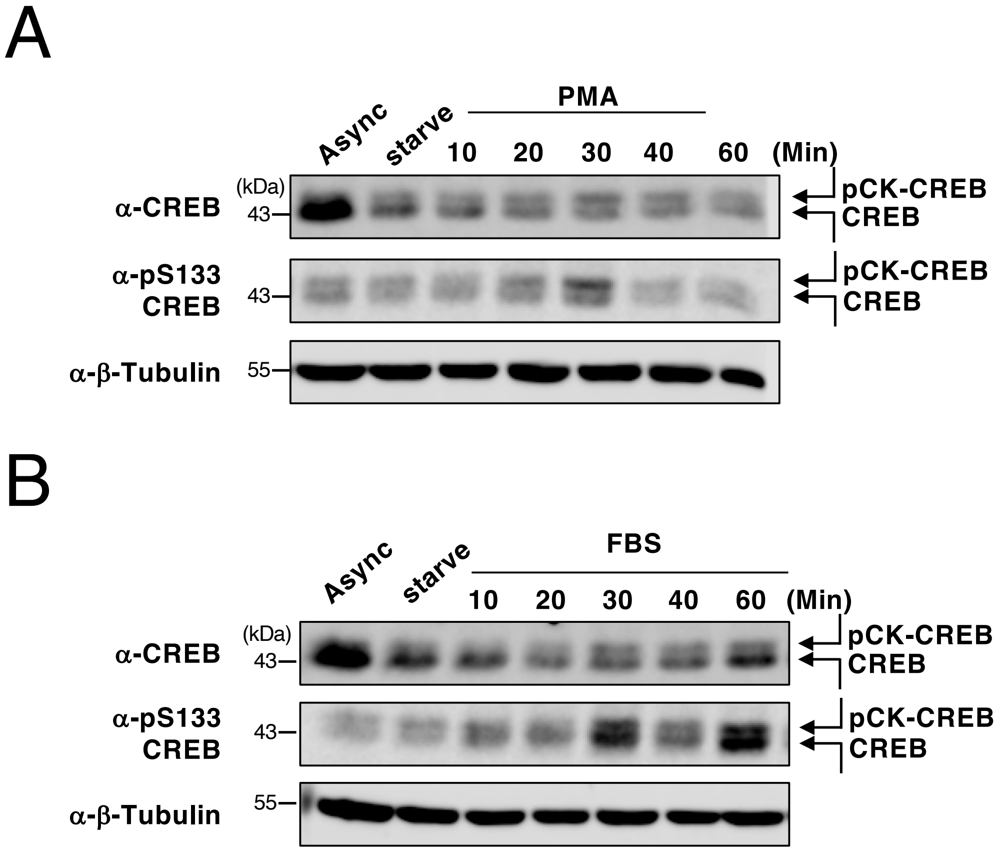

Fig. S1. Measurement of CREB S133 and CK cassette phosphorylation in response to PMA or serum. Serum-starved primary MEFs were stimulated with PMA (200 ng/ml) (A) or 10% FBS (B) for the indicated lengths of time and CK cassette and S133 phosphorylation monitored by Western blotting with α-CREB and α-pS133-CREB antibodies.

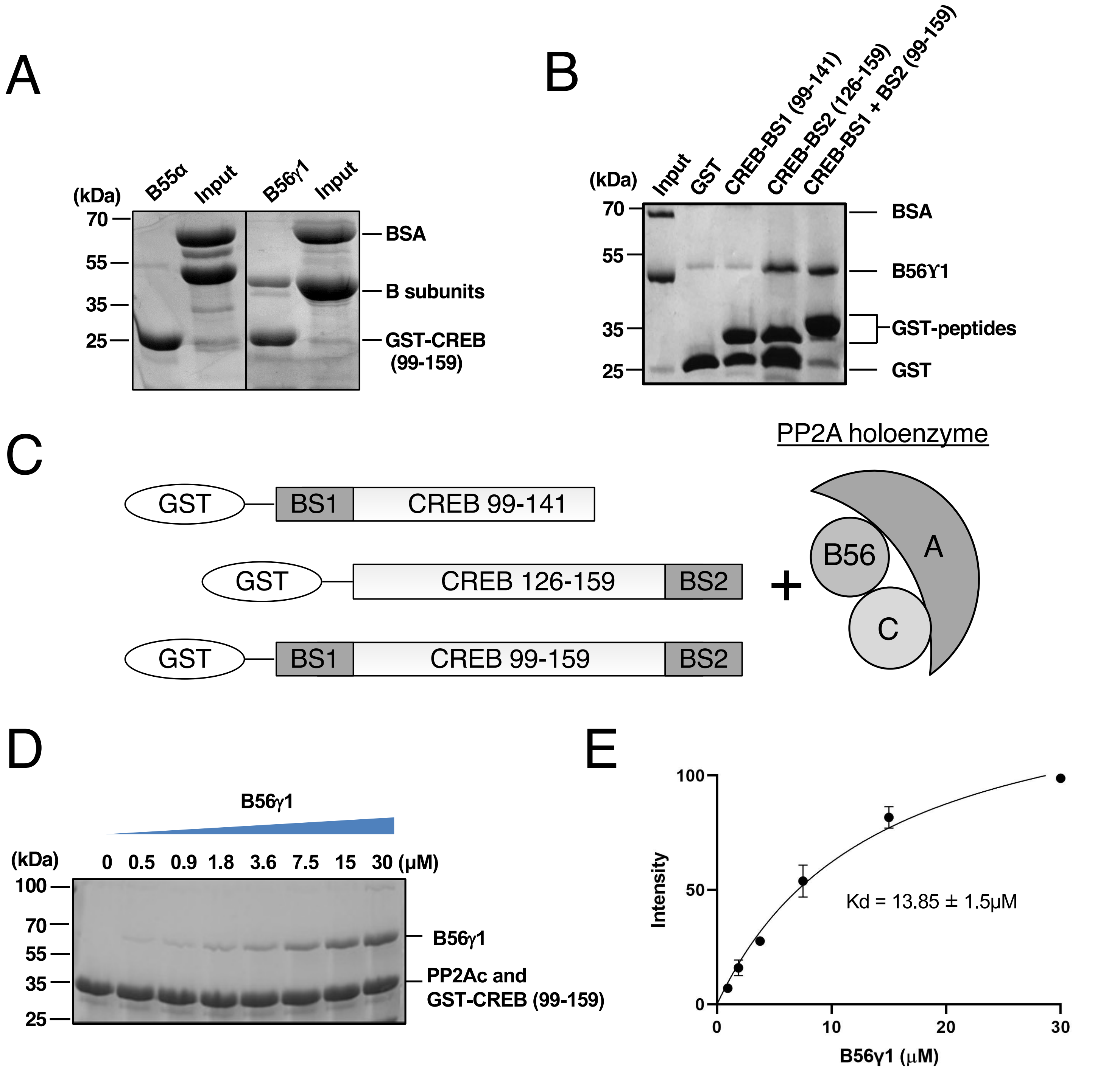

Fig. S2. Binding assays of GST-CREB (99-159) with B55 and B56 subunits. (A) Pull-down of B55α or B56γ1 via GST-CREB (99-159) immobilized on GS4B resin. Bound proteins and input for pulldown were examined on SDS-PAGE and visualized by Coomassie blue staining. **(B)** Pull-down of PP2A-B56γ1 holoenzyme via GST-CREB (99-141), GST-CREB (126-159), or GST-CREB (99-159) immobilized on GS4B resin. Bound proteins and input for pulldown were examined on SDS-PAGE and visualized by Coomassie blue staining. **(C)** Schematic of GST-CREB fusion proteins and PP2A holoenzyme used for pulldown assays. **(D)** Pulldown of GST-CREB (99-159) with titrated concentrations of B56γ1. The bound proteins were examined as in Fig. 2A. **(E)** The intensity of bound PP2Ac was estimated using image J. Results from three independent experiments were used to fit the intensity of bound B56γ1 versus B56γ1 concentrations into one site specific binding model in GraphPad Prism. The Kd is calculated to be 13.85 μM.

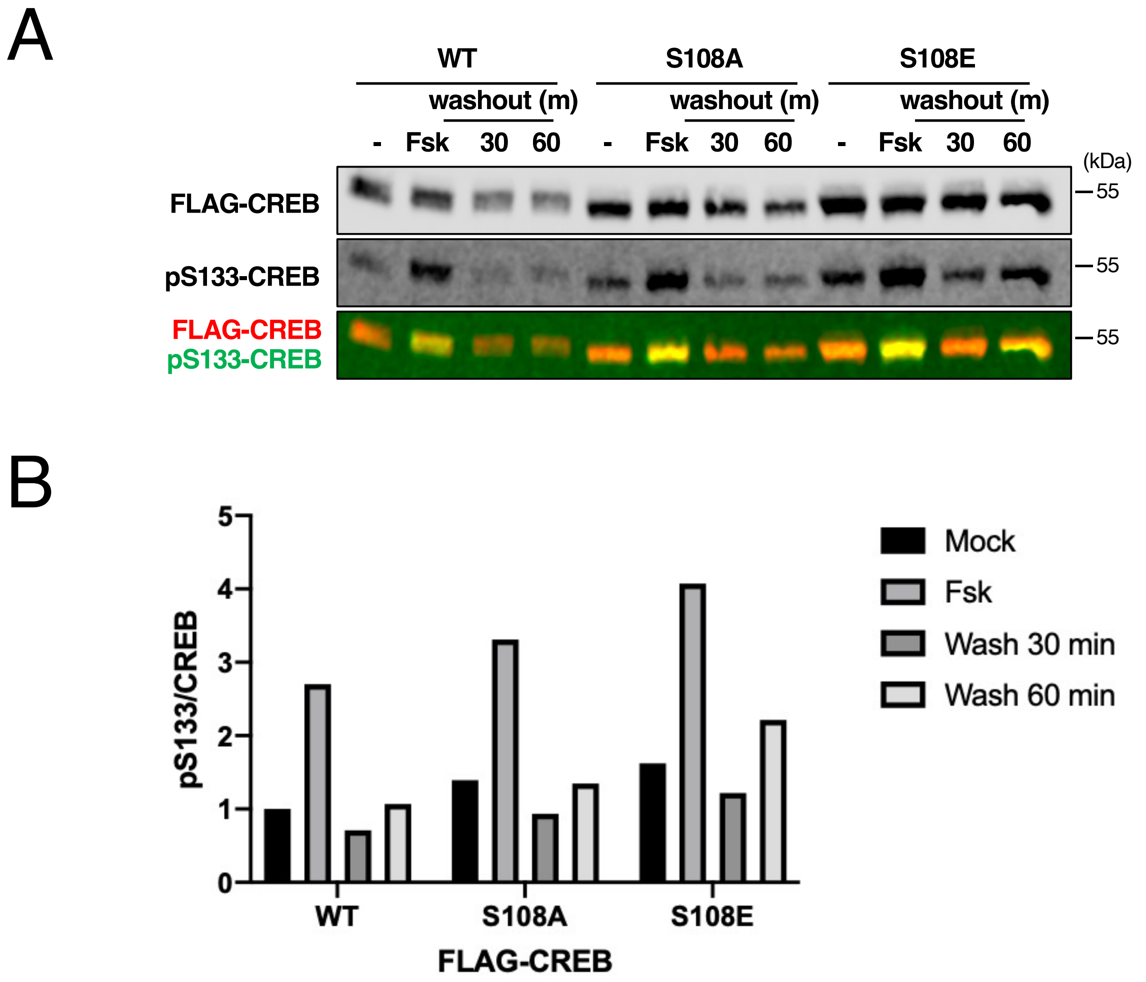

Fig. S3. Effect of phosphomimetic S108E mutation on CREB S133 dephosphorylation. (A) Indicated FLAG-CREB proteins were expressed in HeLa cells which were then treated with Fsk for 30 min prior to drug washout for the indicated lengths of time. Cell extracts were coimmunoblotted with α-FLAG and α-pS133-CREB antibodies. **(B)** Quantification of immunoblotting data from (A).

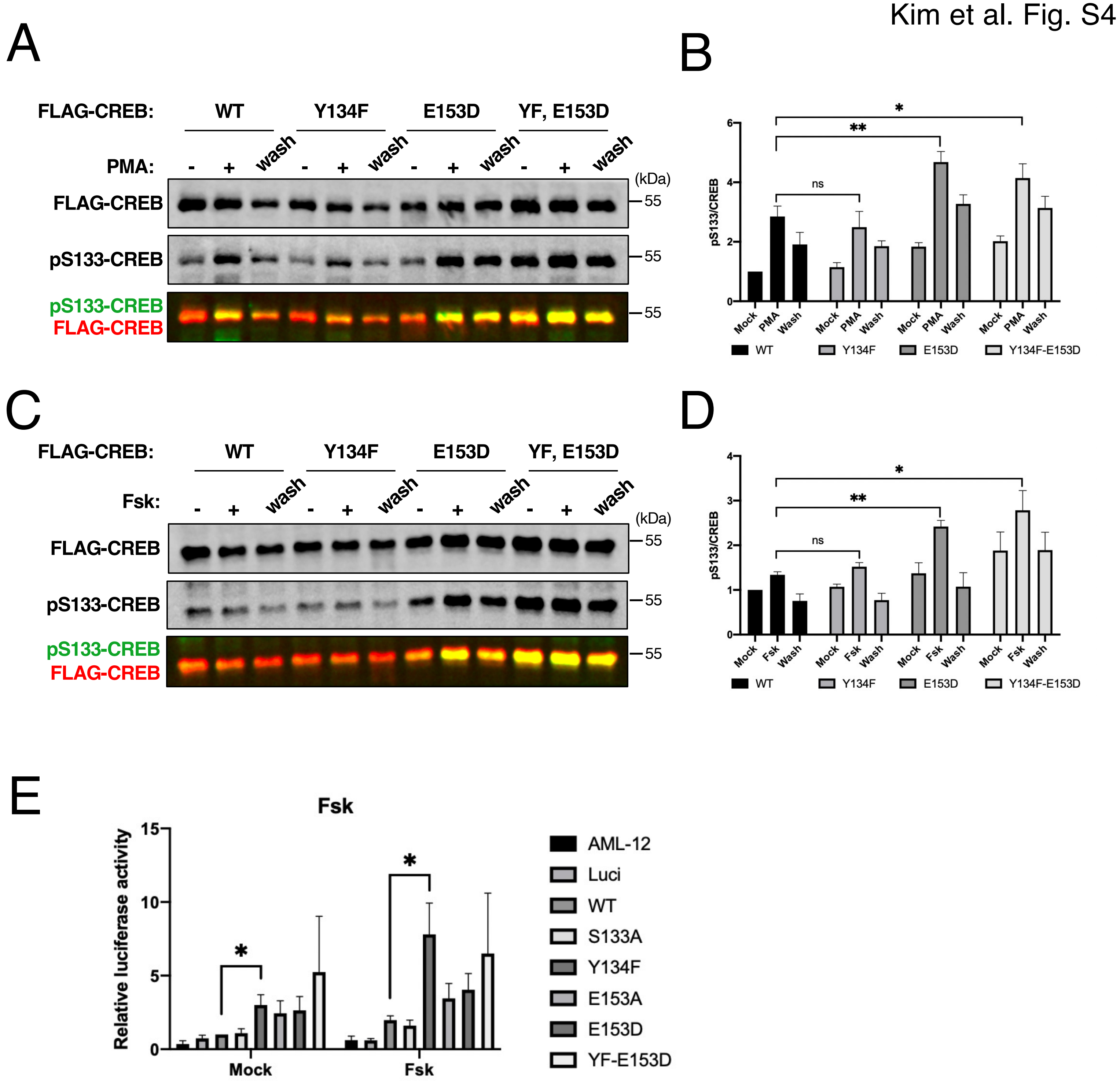

Fig. S4. Relative impacts of Y134F and E153D mutations on CREB S133 phosphorylation. HeLa cells were transfected with indicated FLAG-CREB constructs and exposed to vehicle and either PMA (**A**,**B**) or Fsk (**C**,**D**) followed by an 1 h washout. Cell extracts were coimmunoblotted with α-FLAG and α-pS133 antibodies. Quantification of relative pS133/CREB levels are shown in panels B and D (n=3, *$p < 0.05$; **$p < 0.01$). **(E)** AML-12 hepatocytes were cotransfected with indicated UAS-luciferase (Luc) and Gal4-CREB plasmids. Luciferase activity following serum starvation and Fsk treatment (2 h) is shown (n=3, *$p < 0.05$; **$p < 0.01$; ***$p < 0.005$).

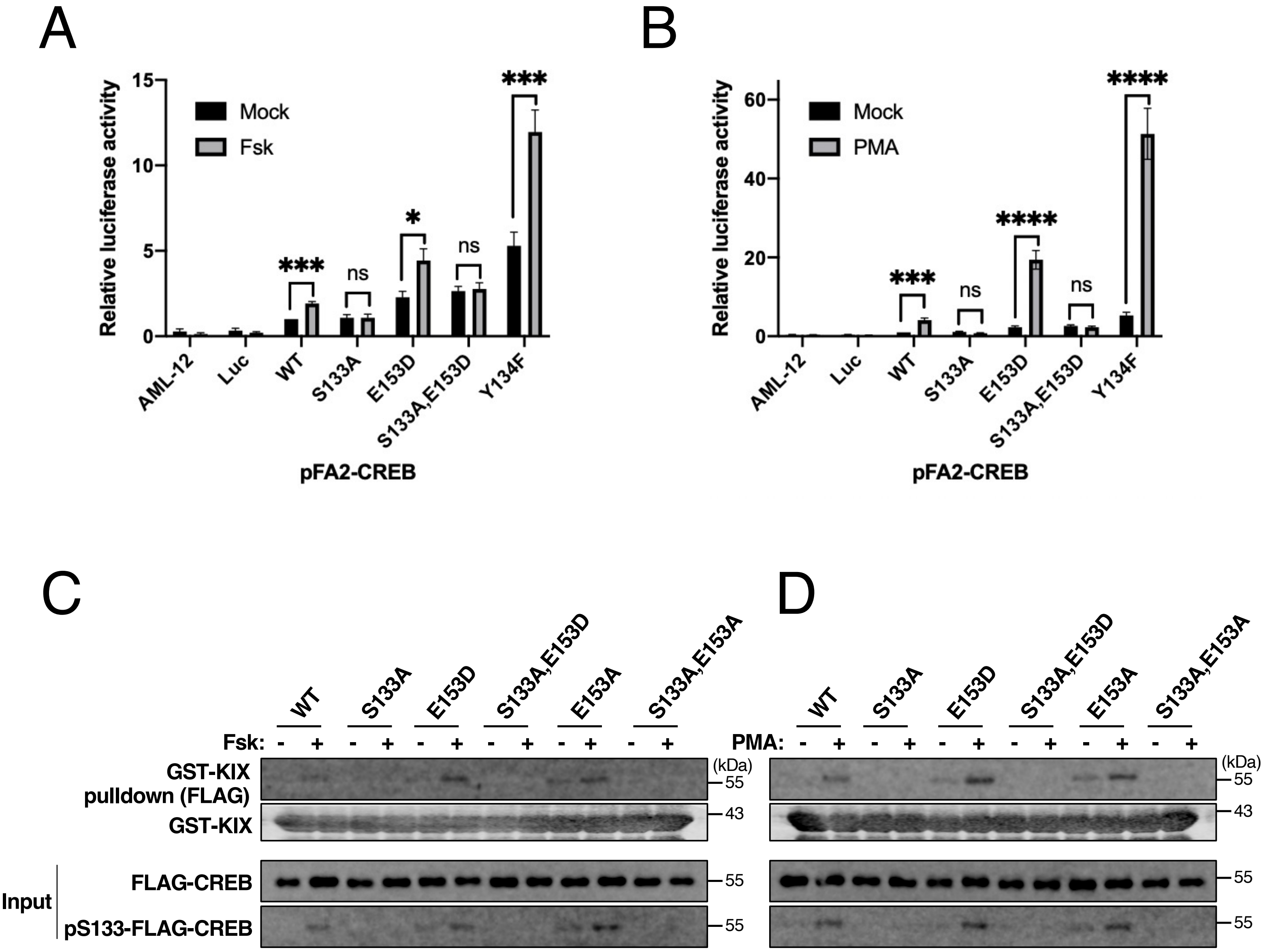

Fig. S5. An S133A mutation reduced CBP binding and transactivation potential of CREB BS2 mutants. (A,B) AML-12 hepatocytes were cotransfected with indicated UAS-Luciferase (Luc) and Gal4-CREB plasmids. Cells were serum starved for 16 h and then treated for 2 h with Fsk (A) or PMA (B) prior to luciferase activity measurements (n=4, *p < 0.05; **p < 0.01; ***p < 0.005; ****p < 0.001). **(C,D)** Indicated FLAG-CREB proteins were expressed in HeLa cells and affinity purified via GST pulldown assays using the KIX domain of CBP. Cells were serum starved for 16 h and then treated for 30 min with Fsk (C) or PMA (D) prior to GST-KIX pulldown assay.

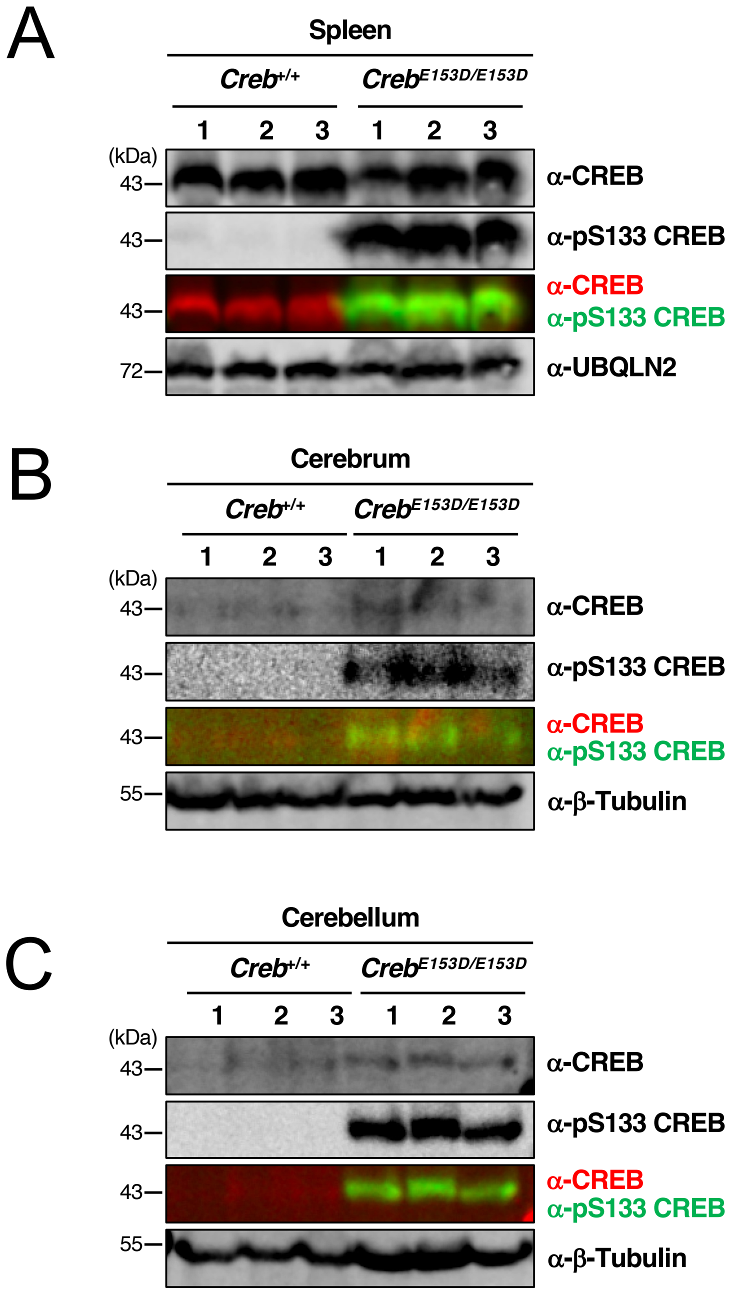

Fig. S6. An endogenous E153D mutation potentiates CREB phosphorylation in brain and spleen. Spleen **(A)** Cerebrum **(B)** Cerebellum **(C)** cell extracts were prepared from 8-week old *Creb*$^{+/+}$ or *Creb*$^{E153D/E153D}$ mice and coimmunoblotted for total CREB and pS133-CREB.
